# Contemporary *Anopheles* vector species distribution in Scotland and its association with historical hotspots of malaria transmission

**DOI:** 10.1186/s12936-026-06061-1

**Published:** 2026-07-19

**Authors:** Kieran Killen, Georgia Kirby, Rebecca Brown, Susanne Krabbendam, Luca Nelli, Heather M. Ferguson

**Affiliations:** School of Biodiversity, One Health and Veterinary Medicine, University of Glasgow, Glasgow, G12 8QQ UK

**Keywords:** Historical malaria, Ague, Mosquitoes, Species distribution models, *Anopheles plumbeus*, *Anopheles maculipennis*, *Anopheles claviger*, Vector surveillance, Scotland

## Abstract

**Background:**

Malaria poses a significant threat to public health in tropical regions, with nearly half of the global population at risk. Historically, transmission was common in temperate regions including northwestern Europe, with records indicating that malaria was prevalent in Scotland until the early nineteenth century. While current environmental and epidemiological conditions are unsuitable for transmission, future climate, environmental, and land-use change may alter the receptivity of Scotland to malaria and other mosquito-borne diseases. As a first step to assess the potential for future transmission risk, we aimed to map the distribution of two native *Anopheles* mosquito species in Scotland that have been implicated in historical malaria transmission and assess the relationship between areas of high contemporary suitability and historical hotspots of transmission.

**Methods:**

Using data generated from a recent Scotland-wide mosquito surveillance programme and citizen science reporting (2023–2024), we used species distribution models to predict the current distribution of *Anopheles claviger* and *An. plumbeus* in Scotland and identify their associations with environmental and land-use variables. Another historical vector, *An. maculipennis* s.l., was also recorded but with too few observations for reliable modelling. Using georeferenced historical parish hotspots of malaria in Scotland (eighteenth century records), we tested whether predicted contemporary suitability at hotspot locations was higher than expected by chance using comparisons against randomly sampled background locations.

**Results:**

Several climatic and physical environmental variables were associated with *Anopheles* species distribution in Scotland, with altitude and landcover being the most consistent, and temperature and precipitation having variable impact across species. *Anopheles* were predicted to have widespread distribution across coastal and lowland Scotland, with pockets of habitat suitability extending up to the northeast coast and Shetland islands. Historical hotspots of malaria (parish locations) were consistently associated with higher predicted current suitability for *Anopheles* species than background locations.

**Conclusions:**

Overall, these findings show that potential malaria vectors are still common throughout Scotland and highlight the need for continued monitoring to generate more accurate estimates of predictors, distribution and future disease risk.

## Introduction

Approximately half of the human population inhabit regions where they are at risk of malaria infection [1, 2]. Caused by *Plasmodium* parasites and spread by female *Anopheles* mosquitoes [3], this disease caused 282 million cases and 610,000 deaths in 2024; with the majority being young African children under the age of five [1]. The current distribution and burden of malaria falls disproportionately in the tropics, but malaria was historically widespread across temperate regions including the United States and numerous European countries [4–7]; with evidence of local transmission extending into polar regions of Finland until the early twentieth century [8]. While malaria was successfully eliminated from North America and Europe in the twentieth century [9, 10], there are concerns that climate change may increase the likelihood of re-establishment in temperate regions [11, 12].

The receptivity of temperate regions to malaria is shaped by a combination of climatic, ecological and socio-economic factors. Temperature influences mosquito development, adult survival and *Plasmodium* development, while precipitation, hydrology and land use affect the availability and persistence of larval habitats [13–25]. However, the historical disappearance of malaria from northern Europe cannot be explained by climate alone. Long-term changes in wetland drainage, agricultural practices, housing quality and broader living conditions, livestock management and stabling practices, availability of overwintering or diapause sites, access to diagnosis and treatment, and reductions in the human parasite reservoir all likely contributed to the interruption of transmission [6, 9, 26, 27]. Therefore, assessing contemporary receptivity requires understanding both the current distribution of competent vectors and the wider ecological and social conditions that would permit transmission. Both historical and contemporary patterns of malaria transmission have been shaped not only by environmental conditions and their effects on *Anopheles* vector populations, but also by socio-economic conditions that determine human exposure and parasite persistence. Long-term environmental and socio-economic changes were pivotal in the elimination of endemic malaria across northern and western Europe during the 19th and early twentieth centuries. Improved housing and living conditions reduced human exposure to biting mosquito vectors, while changes in rural infrastructure, including the loss or altered use of outbuildings, barns and stables that could provide overwintering or diapause sites, together with the drainage of breeding marshes, reduced *Anopheles* persistence. Improved access to diagnosis and treatment also contributed by reducing the human parasite reservoir. Other changes may, however, increase local vector suitability. For example, urbanisation may favour some European vector species like *Anopheles plumbeus* due to its ability to use of artificial containers for oviposition [28, 29]. Additionally, buildings, stables and livestock near human settlements may influence vector populations and human-vector contact in contrasting ways. Built structures can provide resting or overwintering sites for some British *Anopheles* species [21], while urban and peri-urban environments can create artificial aquatic habitats suitable for mosquito development [30]. The effect of livestock on human-vector contact is context dependent, because animal hosts may support mosquito populations while also altering feeding patterns depending on vector host preference, host availability and local husbandry practices. For *An. plumbeus*, abandoned or poorly maintained stables may also provide larval habitats where rainwater accumulates in manure cellars [73]. Many *Anopheles* species are native and widespread across Europe; including at least eight with known competence for malaria (*An. atroparvus, An. sacharovi, An. labranchiae, An. messeae/daciae, An. maculipennis s.s., An. superpictus, An. hyrcanus s.l.* and *An. plumbeus*) [31]. Identifying the environmental determinants of the abundance and distribution of these species is crucial both for understanding historical patterns of transmission and predicting future risks.

The United Kingdom is an example of a northern European country where malaria was historically present [26, 32], but eliminated largely due to changes in land-use and improvements in living conditions [6]. Historically known as ‘Ague’, now thought to be *P. vivax* malaria, this disease was common around marshes in the UK up until the late 1800s [7]. While the quantitative public health burden of during this time is unknown, historical accounts describe residents of these areas as being unhealthy and short-lived due to fevers caused by infection [33, 34]. While historical malaria in the UK is most associated with low lying marshes and fenlands in England and Wales [7, 32], transmission extended into Scotland where records indicate that marsh areas were heavily afflicted until the mid-nineteenth century [35]. In his book published in 1920, James Ritchie concluded based on his analysis of historical records that of all the diseases present in eighteenth century Scotland “*none was more prevalent or more persistent in its attacks and effects*” than the Ague [36]. This is supported by qualitative accounts in parish-level statistical records from the period which highlight significant constraints to agricultural production due to seasonal Ague outbreaks in farm workers (Statistical Accounts of Scotland, 2025 | https://stataccscot.ed.ac.uk). Rapid changes both in land-use and living conditions during the industrial revolution led to the eventual decline of Ague in Scotland [26, 36], with the last case of autochthonous malaria case reported in Scotland occurring in the late summer of 1919 [8]. More recently, imported cases have been regularly reported since the 1960’s [37], with 90 cases identified in 2023 alone [38]. The regular occurrence of imported cases and presence of native vector species in Scotland could thus generate some risk of sporadic transmission or re-introduction should environmental conditions become more favourable in the future.

Three *Anopheles* species are native to Scotland: *An. claviger*, *An. plumbeus* and *An. maculipennis* s.l. [21, 39]. Their relevance to malaria transmission is likely to differ among species. Members of the *An. maculipennis s.l.* complex, particularly *An. atroparvus* elsewhere in Britain and Europe, are generally considered the principal historical malaria vectors. In contrast, *An. claviger* and *An. plumbeus* are better regarded as competent or potential vectors, but there is limited evidence to determine their historical role in malaria transmission in Scotland specifically [29, 40–42]. The ecology, distribution and behaviour of these native taxa may therefore shape future malaria receptivity in Scotland. However, current distributional data for these taxa in Scotland remain limited, with most ecological knowledge coming from studies in England or mainland Europe. Among these taxa, *An. claviger* is the most common throughout the United Kingdom, where it is strongly associated with vegetated wetland habitats [21, 43]. Despite its abundance and *Plasmodium* competence [44], it is unlikely to have been the primary vector of historical malaria due to its zoophilic behaviour [21]. Instead, this is likely to have been *An. maculipennis* s.l., a complex comprising ten morphologically indistinguishable subspecies of which two (*An. atroparvus* and *An. messae*) have been recorded in the UK. Little is known about the presence or distribution of complex members in Scotland, with the few existing records reported generally as *An. maculipennis* s.l. [39, 40]. Like *An. claviger, An. maculipennis* is associated with wetlands but differs somewhat in its ability to use of stagnant, sunlit water bodies [21, 45], artificial larval habitats and brackish coastal waters for oviposition [46, 47]. While *An. maculipennis* s.l. is largely zoophilic, human biting has been documented including during cooler winter months [48]. Finally, *An. plumbeus* is also widely distributed across the UK [39, 49] but is uniquely associated with mature woodlands due to its use of flooded tree holes for larval habitat [29]. *Anopheles plumbeus* is associated with human nuisance biting in other parts of Europe [50, 51], and was responsible for a local malaria outbreak leading to hospitalisations in Germany in the late 1990’s [52].

Assessment of the potential risk posed by these *Anopheles* species in Scotland is unclear due to the lack of current data on their ecology. Assessment of future risk can be facilitated by understanding the current distribution of these vector species, as well as their potential role in historical malaria transmission. Using data from a recent Scotland-wide mosquito surveillance programme (https://www.mosquito-scotland.com [53]), here we aimed to elucidate the current distribution of potential malaria vector species in Scotland and identify the key environmental and land-use factors that underpin it. Additionally, we explored the association between current *Anopheles* vector distribution and historical malaria hotspots in Scotland. In combination, results will support the assessment of potential risks of sporadic malaria transmission in Scotland under current or future environmental conditions, and guide spatially-explicit identification of priority sites for surveillance.

## Methods

### Data collation

Data on the contemporary occurrence of the three target malaria vector species in Scotland (*An. claviger, An. plumbeus,* and *An. maculipennis s.l*) was collected from three primary sources. The first was data from the Mosquito Scotland Project (https://www.mosquito-scotland.com), through which data were obtained from mosquito surveys conducted at 22 spatially representative wetlands across Scotland between June and October 2023 [53], and data from long term sampling at 3 sites (WWT Caerlaverock, Scottish Centre for Ecology and Hydrology, and Montrose Basin) in Scotland between April and September 2024. The Mosquito Scotland project also contributed citizen science data on mosqito observations and bites that were submitted to a dedicated reporting system (https://www.mosquito-scotland.com/submit-a-mosquito). Specimens or photos submitted through this site that could be identified to *Anopheles* species level were also included in the data, as well as records from reactive mosquito sampling carried out by the Mosquito Scotland team in response to clusters of citizen science reports at some locations in 2024 (G. Kirby, pers comm). Other *Anopheles* data were obtained from the National Biodiversity Network [54] and two recently published datasets with additional mosquito records for Scotland [49]. A csv spreadsheet was created in Microsoft Excel where each observation point was allocated a row, with the associated coordinate centroids, date (ranging between 1909 and 2024) and species of *Anopheles* also being included.

### Environmental predictors

We modelled species occurrence as a function of topographic, climatic, land cover, hydrological and human population-density. Predictors were summarised within a 3-km circular buffer around each observation (presence and pseudoabsence – see following paragraphs) and included altitude (ALT, obtained from a digital elevation model with a spatial resolution of 50 m, downloaded from www.data.gov.uk); human population density within 3 km (POP, extracted from WorldPop gridded population surfaces ([55], www.worldpop.org); proportional land-cover classes within 3 km derived from the UK Centre for Ecology and Hydrology ([56], www.ceh.ac.uk); and climatic covariates comprising precipitation (PREC), air temperature (TEMP) and relative humidity (HUM) obtained from NASA Langley’s Prediction Of Worldwide Energy Resources (POWER) data services [57]. To reduce dimensionality and mitigate overfitting risk, the 21 UKCEH land-cover classes were aggregated into 10 broader classes following the UKCEH aggregation framework (Table 1), and the proportion of each aggregated class within each 3-km buffer was calculated and used as model inputs.

**Table 1:** UKCEH land-use classes and the 10 classes they can be aggregated into

Original UKCEH land-use class	Aggregated variable (Proportion in 3 km buffer)
Broadleaf woodland	${P}_{\mathrm{B}\mathrm{r}\mathrm{o}\mathrm{a}\mathrm{d}\mathrm{l}\mathrm{e}\mathrm{a}\mathrm{f}}$
Coniferous woodland	${P}_{\mathrm{C}\mathrm{o}\mathrm{n}\mathrm{i}\mathrm{f}\mathrm{e}\mathrm{r}}$
Arable and horticulture	${P}_{\mathrm{A}\mathrm{r}\mathrm{a}\mathrm{b}\mathrm{l}\mathrm{e}}$
Improved grassland	${P}_{\mathrm{I}\mathrm{m}\mathrm{p}\mathrm{G}\mathrm{r}\mathrm{a}\mathrm{s}\mathrm{s}}$
Neutral grassland	${P}_{\mathrm{S}\mathrm{e}\mathrm{m}\mathrm{i}\mathrm{N}\mathrm{a}\mathrm{t}\mathrm{G}\mathrm{r}\mathrm{a}\mathrm{s}\mathrm{s}}$
Calcareous grassland	
Acid grassland	
Fen, marsh and swamp	
Heather	${P}_{\mathrm{M}\mathrm{H}\mathrm{B}\mathrm{G}\mathrm{r}\mathrm{a}\mathrm{s}\mathrm{s}}$
Heather grassland	
Bog	
Inland rock	
Saltwater	${P}_{\mathrm{S}\mathrm{a}\mathrm{l}\mathrm{t}\mathrm{w}\mathrm{a}\mathrm{t}\mathrm{e}\mathrm{r}}$
Freshwater	${P}_{\mathrm{F}\mathrm{r}\mathrm{e}\mathrm{s}\mathrm{h}\mathrm{w}\mathrm{a}\mathrm{t}\mathrm{e}\mathrm{r}}$
Supra-littoral rock	${P}_{\mathrm{C}\mathrm{o}\mathrm{a}\mathrm{s}\mathrm{t}\mathrm{a}\mathrm{l}}$
Supra-littoral sediment	
Littoral rock	
Littoral sediment	
Saltmarsh	
Urban	${P}_{\mathrm{B}\mathrm{u}\mathrm{i}\mathrm{l}\mathrm{t}\mathrm{U}\mathrm{p}}$
Suburban	

The extracted land-use values in this investigation were in the form of the aggregate classes

### Pseudoabsence generation and sampling bias correction

For each species-specific model, we generated pseudoabsence locations at a 10:1 ratio relative to presences (ten pseudoabsences per presence) to characterise the available environmental background for presence–pseudoabsence modelling. Pseudoabsences were not sampled uniformly across Scotland because opportunistic occurrence data are typically affected by sample selection bias, whereby records are concentrated in accessible or frequently visited areas. If background points are sampled from the full landscape, this mismatch can bias inference and inflate apparent model performance [58]. Instead, we used kernel density estimation (KDE) of presence locations to construct a spatial sampling-intensity surface and then drew pseudoabsences probabilistically from this surface, such that pseudoabsences were more likely to be placed in areas with higher estimated sampling intensity. This “bias-informed background” approach aims to better align the spatial bias structure of background samples with that of the occurrence data, reducing confounding between habitat suitability and sampling effort [58–60]

### Species distribution modelling

We initially compiled occurrence records for three *Anopheles* species (*An. claviger*, *An. plumbeus* and *An. maculipennis*); however, *An. maculipennis* was excluded from species distribution modelling due to the small number of presence records (n = 5), which was insufficient to support stable estimation of smooth effects and spatial structure. We therefore fitted separate species-specific Generalised Additive Models (GAMs) for *An. claviger* and *An. plumbeus* using separate species-specific Generalised Additive Models (GAMs) with a binomial error distribution and logit link, fitted under a presence–pseudoabsence design. For each species $s\in \{claviger,plumbeus\}$, let ${o}_{i,s}\in \{\mathrm{0,1}\}$ denote occurrence at location i (1 = presence, 0 = pseudoabsence), with spatial coordinates $\left({x}_{i},{y}_{i}\right)$. We assumed:

$${o}_{i,s}\sim \mathrm{B}\mathrm{e}\mathrm{r}\mathrm{n}\mathrm{o}\mathrm{u}\mathrm{l}\mathrm{l}\mathrm{i}({p}_{i,s}),$$

$$\mathrm{l}\mathrm{o}\mathrm{g}\mathrm{i}\mathrm{t}({p}_{i,s})={\beta}_{0,s}+{f}_{ALT,s}({\mathrm{A}\mathrm{L}\mathrm{T}}_{i})+{f}_{POP,s}({\mathrm{P}\mathrm{O}\mathrm{P}}_{3\mathrm{k}\mathrm{m},i})+\sum_{c\in \mathcal{L}}{f}_{c,s}({c}_{i})+{f}_{PREC,s}({\mathrm{P}\mathrm{R}\mathrm{E}\mathrm{C}}_{i})+{f}_{TEMP,s}({\mathrm{T}\mathrm{E}\mathrm{M}\mathrm{P}}_{i})+{f}_{HUM,s}({\mathrm{H}\mathrm{U}\mathrm{M}}_{i})+{f}_{XY,s}({x}_{i},{y}_{i}),$$

where ${\beta}_{0,s}$ is a species-specific intercept and each ${f}_{\cdot ,s}(\cdot )$ is a smooth function estimated from the data. The land-cover set $\mathcal{L}$ comprised proportional cover within 3 km of: arable (${P}_{\mathrm{A}\mathrm{r}\mathrm{a}\mathrm{b}\mathrm{l}\mathrm{e}}$), improved grassland (${P}_{\mathrm{I}\mathrm{m}\mathrm{p}\mathrm{G}\mathrm{r}\mathrm{a}\mathrm{s}\mathrm{s}}$), semi-natural grassland (${P}_{\mathrm{S}\mathrm{e}\mathrm{m}\mathrm{i}\mathrm{N}\mathrm{a}\mathrm{t}\mathrm{G}\mathrm{r}\mathrm{a}\mathrm{s}\mathrm{s}}$), mountain/heath/bog/grassland (${P}_{\mathrm{M}\mathrm{H}\mathrm{B}\mathrm{G}\mathrm{r}\mathrm{a}\mathrm{s}\mathrm{s}}$), saltwater (${P}_{\mathrm{S}\mathrm{a}\mathrm{l}\mathrm{t}\mathrm{w}\mathrm{a}\mathrm{t}\mathrm{e}\mathrm{r}}$), freshwater (${P}_{\mathrm{F}\mathrm{r}\mathrm{e}\mathrm{s}\mathrm{h}\mathrm{w}\mathrm{a}\mathrm{t}\mathrm{e}\mathrm{r}}$), coastal (${P}_{\mathrm{C}\mathrm{o}\mathrm{a}\mathrm{s}\mathrm{t}\mathrm{a}\mathrm{l}}$), and built-up (${P}_{\mathrm{B}\mathrm{u}\mathrm{i}\mathrm{l}\mathrm{t}\mathrm{U}\mathrm{p}}$). Climate covariates were precipitation (PREC), temperature (TEMP), and humidity (HUM).

A bivariate spatial smooth ${f}_{XY,s}({x}_{i},{y}_{i})$ was included to capture residual spatial structure not explained by measured environmental predictors. The spatial smooth was fitted with a restricted basis dimension (k=20) to limit complexity and reduce the risk of overfitting fine-scale spatial patterns. All smooth terms were estimated under penalised likelihood, with smoothing parameters selected by REML; shrinkage selection was enabled so that weak covariate effects could be penalised towards zero during model fitting. Models were fitted separately for each species using only records assigned to that species.

Model discrimination was assessed using the area under the receiver operating characteristic curve (ROC–AUC), interpreted as the probability that a randomly selected presence location receives a higher predicted suitability than a randomly selected pseudoabsence location [61]. As conventional random cross-validation can yield optimistic estimates when observations are spatially clustered or otherwise structured, we prioritised spatially blocked fivefold cross-validation [62]. Observations were partitioned into approximately 30 km spatial blocks and models were iteratively fitted on four folds while evaluating predictions on the held-out fold; performance was summarised as the mean and standard deviation of ROC–AUC across folds [63]. For comparison, we also calculated in-sample AUC and random fivefold cross-validation AUC as secondary diagnostics. ROC curves and AUC were computed using the pROC package in R [64], and spatial blocking was implemented using blockCV [63].

### Habitat suitability prediction and mapping

Spatial predictions of habitat suitability were generated by applying each fitted species-specific GAM to a regular prediction grid covering Scotland at 1 km × 1 km resolution. For each grid cell g with coordinates $\left({x}_{g},{y}_{g}\right)$ and associated covariate vector ${\mathbf{z}}_{g}$, we computed the predicted probability of occurrence (interpreted as relative habitat suitability under the presence–pseudoabsence design) as

$${\widehat{p}}_{s}(g)\hspace{0.25em} =\hspace{0.25em} {logit}^{-1}\hspace{0.17em}\left({\widehat{\beta }}_{0,s}\hspace{0.25em} +\hspace{0.25em} \sum_{m}{\widehat{f}}_{m,s}({z}_{gm})\hspace{0.25em} +\hspace{0.25em} {\widehat{f}}_{XY,s}({x}_{g},{y}_{g})\right),$$

where $s\in \{claviger,plumbeus\}$. Predicted values were then rasterised onto the 1 km grid to create continuous suitability surfaces for each species across Scotland.

To summarise areas suitable for either focal vector species, we derived a combined suitability surface by taking the cell-wise maximum of the two species-specific predictions:

$${\widehat{p}}_{\mathrm{m}\mathrm{a}\mathrm{x}}(g)\hspace{0.25em} =\hspace{0.25em} \mathrm{m}\mathrm{a}\mathrm{x}\left({\widehat{p}}_{\mathrm{c}\mathrm{l}\mathrm{a}\mathrm{v}\mathrm{i}\mathrm{g}\mathrm{e}\mathrm{r}}(g),\hspace{0.25em} {\widehat{p}}_{\mathrm{p}\mathrm{l}\mathrm{u}\mathrm{m}\mathrm{b}\mathrm{e}\mathrm{u}\mathrm{s}}(g)\right).$$

All prediction and mapping steps were performed in a consistent coordinate reference system, and rasters were visualised as continuous suitability maps for each species and for the combined surface.

### Historical malaria concordance analysis

Historical malaria (“ague”) case locations in Scotland were collated from a map created and published by James Ritchie in his 1920 book “*The Influence of Man on Animal Life in Scotland*” [36]. This map consisted of points representing parishes in eighteenth century Scotland where records indicated that “ague was common” based on Ritchie’s qualitative analyses of these written accounts. No reference is made to the names of parishes, nor is an exact time frame or number of cases provided as supplement to this map.

Parishes identified by Ritchie [35] as ague hotspots were digitised from the published map and treated as historical administrative units rather than direct equivalents of modern Scottish administrative boundaries. Because parish boundaries and names have changed over time, and because Ritchie’s synthesis reports hotspots at the parish level (rather than as precise case coordinates), we operationalised each hotspot as an approximate point location. Specifically, the map figure was georeferenced in QGIS by matching stable coastline and river confluences to modern basemap features, and the hotspot symbols were then manually digitised as a point shapefile placed at the mapped parish hotspot location (i.e., the position indicated by Ritchie’s map, not a modern parish centroid). This procedure yields a spatial representation of hotspot locations at the resolution of historical parishes, which vary substantially in area and can span several to many kilometres; consequently, hotspot locations should be interpreted as approximate parish-level indicators rather than exact case locations. Digitised points were filtered to exclude records lacking spatial information and any falling outside the terrestrial prediction mask (i.e., raster cells with missing suitability). To assess whether historical case locations were associated with higher contemporary vector habitat suitability than expected by chance, we extracted predicted suitability values at each historical case location from (i) the *An. claviger* suitability surface ${\widehat{p}}_{\mathrm{c}\mathrm{l}\mathrm{a}\mathrm{v}\mathrm{i}\mathrm{g}\mathrm{e}\mathrm{r}}(g)$, (ii) the *An. plumbeus* surface ${\widehat{p}}_{\mathrm{p}\mathrm{l}\mathrm{u}\mathrm{m}\mathrm{b}\mathrm{e}\mathrm{u}\mathrm{s}}(g)$, and (iii) the combined surface ${\widehat{p}}_{\mathrm{m}\mathrm{a}\mathrm{x}}(g)=\mathrm{m}\mathrm{a}\mathrm{x}({\widehat{p}}_{\mathrm{c}\mathrm{l}\mathrm{a}\mathrm{v}\mathrm{i}\mathrm{g}\mathrm{e}\mathrm{r}}(g),{\widehat{p}}_{\mathrm{p}\mathrm{l}\mathrm{u}\mathrm{m}\mathrm{b}\mathrm{e}\mathrm{u}\mathrm{s}}(g))$. For each surface, we compared the distribution of suitability values at historical case locations to a background distribution generated by sampling a large set of random locations from the corresponding prediction mask (excluding cells with missing predictions). Statistical support for an association was quantified using a one-sided Wilcoxon rank-sum test (alternative hypothesis: suitability at historical locations exceeds background suitability) and an AUC-like probability-of-superiority statistic, defined as the probability that a randomly selected historical location has higher predicted suitability than a randomly selected background location (ties assigned half weight). As an additional summary, we estimated a permutation-based p-value for mean suitability at historical locations by repeatedly resampling background suitability values (with replacement) to match the number of historical cases. As a secondary, threshold-based metric for communication, we calculated the proportion of historical cases in “high suitability” areas and compared it to the background proportion (ratio of proportions). Species-specific thresholds were derived using ROC-based criteria applied to contemporary occurrence vs pseudoabsence predictions (maximising sensitivity + specificity). For the combined analysis, “high suitability for at least one vector species” was defined as locations where ${\widehat{p}}_{\mathrm{c}\mathrm{l}\mathrm{a}\mathrm{v}\mathrm{i}\mathrm{g}\mathrm{e}\mathrm{r}}\ge 0.180$ or ${\widehat{p}}_{\mathrm{p}\mathrm{l}\mathrm{u}\mathrm{m}\mathrm{b}\mathrm{e}\mathrm{u}\mathrm{s}}\ge 0.155$.

## Results

The collated dataset comprised 364 female *Anopheles* observations identified to one of the three focal taxa: 275 *An. claviger*, 68 *An. plumbeus* and 21 *An. maculipennis s.l.* (Table 2). Records were obtained from three main sources: the Mosquito Scotland project, which contributed 98 female observations; the National Biodiversity Network (NBN), which contributed 147 observations; and published datasets, which contributed 119 observations. Mosquito Scotland records included structured wetland surveys, long-term monitoring, reactive sampling and citizen-science submissions; citizen-science records were retained only when submitted specimens or photographs allowed reliable identification to *Anopheles* species level. *Anopheles claviger* was the most frequently recorded species across all data sources, whereas *An. maculipennis s.l.* was comparatively rare.

**Table 2:** Number of female *Anopheles* observations by species and data source

Data source	*An. claviger* females	*An. maculipennis s.l.* females	*An. plumbeus* females	Total
Mosquito Scotland	65	4	29	98
NBN	113	9	25	147
Published datasets	97	8	14	119
**Total**	**275**	**21**	**68**	**364**

**Fig. 1:**
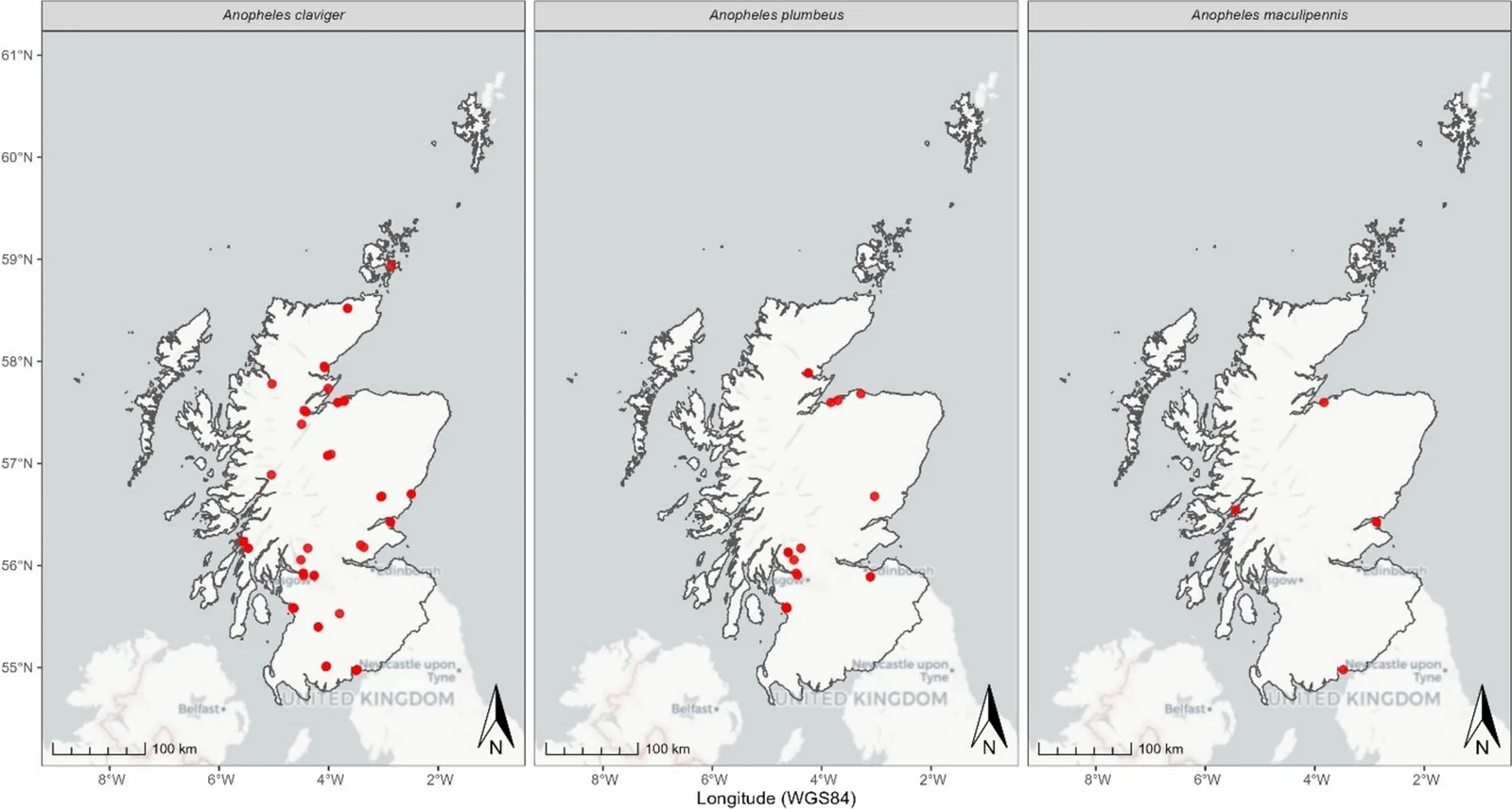
Occurrence data (presence only) for *Anopheles claviger*, *Anopheles plumbeus*, and *Anopheles maculipennis* in Scotland

For species distribution modelling, observations were processed into species-specific occurrence records suitable for spatial analysis, including removal of duplicate or otherwise unsuitable records where required. This resulted in 65 occurrence records for *An. claviger*, 25 for *An. plumbeus* and 5 for *An. maculipennis s.l.* (Fig. 1). Occurrence records covered much of mainland Scotland, with both focal species detected widely across the mainland and a single additional record of *An. claviger* from Orkney.

Separate GAMs fitted to presence–pseudoabsence data explained substantial variation in occurrence for both focal species, with an explained deviance of 43.8% for *An. claviger* (n = 715; Table 3) and 53.8% for *An. plumbeus* (n = 275; Table 4). Model discrimination was high in in-sample evaluation (ROC–AUC = 0.936 for *An. claviger*; 0.956 for *An. plumbeus*) and remained strong under random fivefold cross-validation (mean ROC–AUC = 0.830 ± 0.044 for *An. claviger*; 0.928 ± 0.029 for *An. plumbeus*). Under spatially blocked fivefold cross-validation using 30 km blocks—our primary estimate of spatial transferability—discrimination decreased as expected but remained moderate-to-high (mean ROC–AUC = 0.746 ± 0.094 for *An. claviger*; 0.907 ± 0.047 for *An. plumbeus*). Collectively, these results indicate stronger and more spatially transferable discrimination for *An. plumbeus*, whereas *An. claviger* performance was more variable among spatial folds, consistent with stronger residual spatial structure in the fitted model.

**Table 3:** Summary of the binomial GAM for *Anopheles claviger* occurrence fitted to presence–pseudoabsence data (logit link)

Term	Estimate / edf	Std. Error / Ref.df	z value / Chi-sq	p-value
Parametric coefficients				
(Intercept)	−4.19	0.652	−6.436	< 0.001
Smooth terms				
s(ALT)	9.45 × 10^–1^	9	17.186	< 0.001
s(POP)	6.78 × 10^–6^	9	0.000	0.830
$s({P}_{\mathrm{A}\mathrm{r}\mathrm{a}\mathrm{b}\mathrm{l}\mathrm{e}})$	2.31	9	10.324	0.002
$s({P}_{\mathrm{I}\mathrm{m}\mathrm{p}\mathrm{G}\mathrm{r}\mathrm{a}\mathrm{s}\mathrm{s}})$	2.13	9	9.133	0.004
$s({P}_{\mathrm{S}\mathrm{e}\mathrm{m}\mathrm{i}\mathrm{N}\mathrm{a}\mathrm{t}\mathrm{G}\mathrm{r}\mathrm{a}\mathrm{s}\mathrm{s}})$	6.50 × 10^–6^	9	0.000	0.648
$s({P}_{\mathrm{M}\mathrm{H}\mathrm{B}\mathrm{G}\mathrm{r}\mathrm{a}\mathrm{s}\mathrm{s}})$	6.77 × 10^–1^	9	2.095	0.064
$s({P}_{\mathrm{S}\mathrm{a}\mathrm{l}\mathrm{t}\mathrm{w}\mathrm{a}\mathrm{t}\mathrm{e}\mathrm{r}})$	7.98 × 10^–1^	9	3.940	0.022
$s({P}_{\mathrm{F}\mathrm{r}\mathrm{e}\mathrm{s}\mathrm{h}\mathrm{w}\mathrm{a}\mathrm{t}\mathrm{e}\mathrm{r}})$	1.17	9	3.716	0.034
$s({P}_{\mathrm{C}\mathrm{o}\mathrm{a}\mathrm{s}\mathrm{t}\mathrm{a}\mathrm{l}})$	9.76 × 10^–1^	9	2.481	0.060
$s({P}_{\mathrm{B}\mathrm{u}\mathrm{i}\mathrm{l}\mathrm{t}\mathrm{U}\mathrm{p}})$	1.50 × 10^-5^	9	0.000	0.270
s(PREC)	1.68	9	4.213	0.005
s(TEMP)	2.93	9	7.853	0.002
s(HUM)	8.42 × 10^–1^	9	5.326	< 0.001
s(X,Y)	11.21	19	35.007	< 0.001

Reported terms include estimated degrees of freedom (edf), test statistics and approximate significance for each smooth, along with sample size, deviance explained and adjusted ${R}^{2}$

**Table 4:** Summary of the binomial GAM for *Anopheles plumbeus* occurrence fitted to presence–pseudoabsence data (logit link)

Term	Estimate / edf	Std. Error / Ref.df	z value / Chi-sq	p-value
Parametric coefficients				
(Intercept)	−10.21	3.754	−2.719	0.007
Smooth terms				
s(ALT)	9.35 × 10^–1^	8	14.300	< 0.001
s(POP)	6.24 × 10^–6^	9	0.000	0.445
${s(P}_{\mathrm{A}\mathrm{r}\mathrm{a}\mathrm{b}\mathrm{l}\mathrm{e}})$	5.28 × 10^–6^	9	0.000	0.463
$s({P}_{\mathrm{I}\mathrm{m}\mathrm{p}\mathrm{G}\mathrm{r}\mathrm{a}\mathrm{s}\mathrm{s}})$	7.62 × 10^–1^	9	3.210	0.036
$s({P}_{\mathrm{S}\mathrm{e}\mathrm{m}\mathrm{i}\mathrm{N}\mathrm{a}\mathrm{t}\mathrm{G}\mathrm{r}\mathrm{a}\mathrm{s}\mathrm{s}})$	1.35	8	4.610	0.036
$s({P}_{\mathrm{M}\mathrm{H}\mathrm{B}\mathrm{G}\mathrm{r}\mathrm{a}\mathrm{s}\mathrm{s}})$	4.06 × 10^–6^	9	0.000	0.428
$s({P}_{\mathrm{S}\mathrm{a}\mathrm{l}\mathrm{t}\mathrm{w}\mathrm{a}\mathrm{t}\mathrm{e}\mathrm{r}})$	1.28 × 10^–5^	9	0.000	0.304
$s({P}_{\mathrm{F}\mathrm{r}\mathrm{e}\mathrm{s}\mathrm{h}\mathrm{w}\mathrm{a}\mathrm{t}\mathrm{e}\mathrm{r}})$	7.45 × 10^–1^	9	2.924	0.040
$s({P}_{\mathrm{C}\mathrm{o}\mathrm{a}\mathrm{s}\mathrm{t}\mathrm{a}\mathrm{l}})$	9.61 × 10^–1^	9	1.808	0.141
$s({P}_{\mathrm{B}\mathrm{u}\mathrm{i}\mathrm{l}\mathrm{t}\mathrm{U}\mathrm{p}})$	8.87 × 10^–6^	9	0.000	0.388
s(PREC)	2.23 × 10^–6^	9	0.000	0.720
s(TEMP)	3.71 × 10^–6^	9	0.000	0.537
s(HUM)	2.49 × 10^–6^	9	0.000	0.575
s(X,Y)	2.49 × 10^–6^	19	0.000	0.892

Reported terms include estimated degrees of freedom (edf), test statistics and approximate significance for each smooth, along with sample size, deviance explained and adjusted ${R}^{2}$. Shrinkage selection penalised weak terms towards zero (edf ≈ 0)

For *An. claviger*, multiple environmental predictors were retained as informative, including altitude, land-cover composition (arable, improved grassland, freshwater and saltwater), and climatic variables (precipitation, temperature and humidity), alongside a pronounced residual spatial component (Table 3). In contrast, the *An. plumbeus* model was more parsimonious after shrinkage, with strongest support for altitude and a subset of land-cover/hydrology predictors (improved grassland, semi-natural grassland, freshwater), and little evidence of additional residual spatial structure beyond measured covariates (Table 4).

**Fig. 2:**
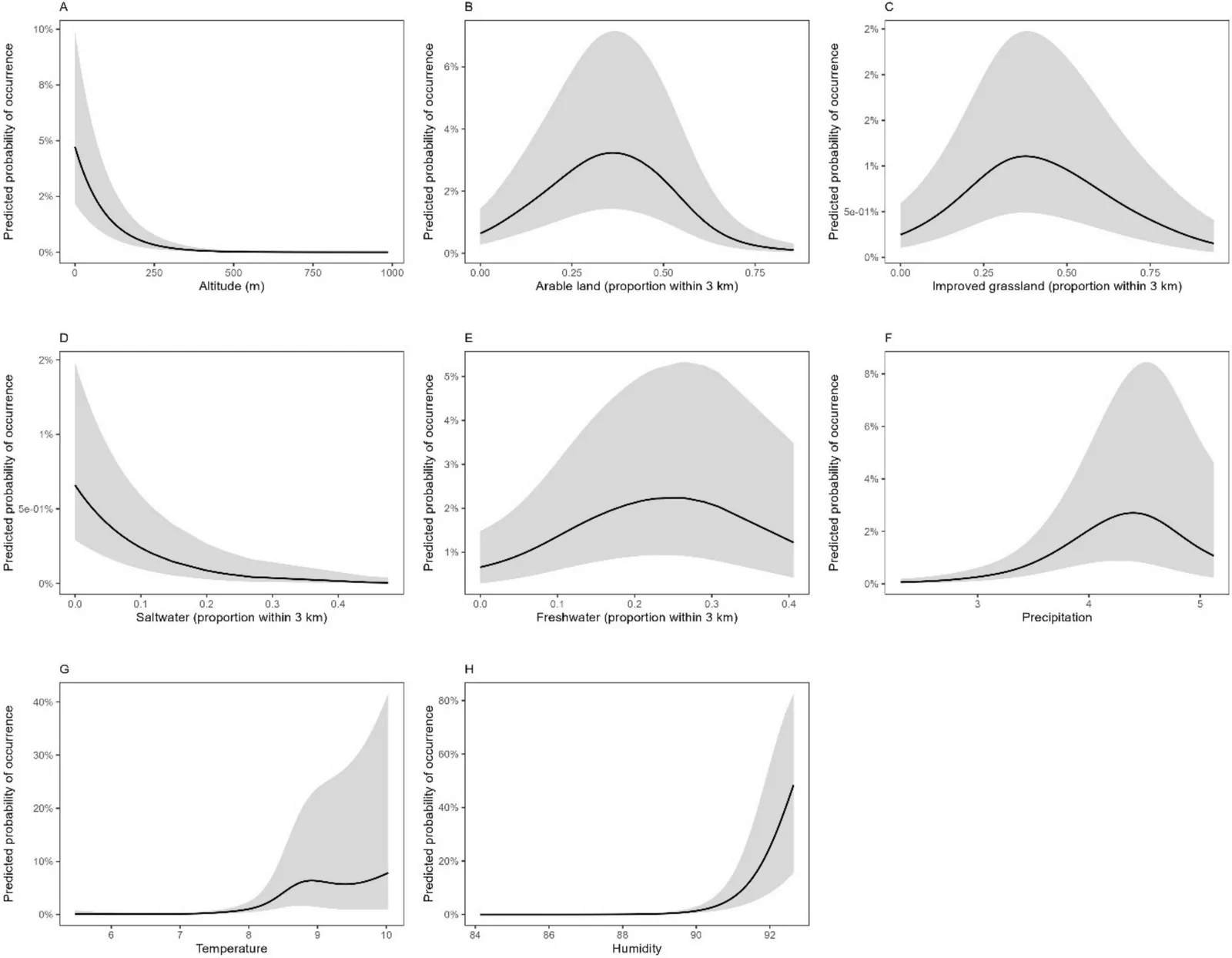
Partial effect plots from the *Anopheles claviger* GAM showing the marginal association between predicted response-scale values (interpreted as relative habitat suitability under the presence–pseudoabsence design) and each retained environmental predictor: **A** altitude, **B** arable land, **C** improved grassland, **D** saltwater, **E** freshwater, **F** precipitation, **G** temperature, **H** humidity. Solid lines show fitted effects and shaded ribbons indicate 95% confidence intervals

**Fig. 3:**
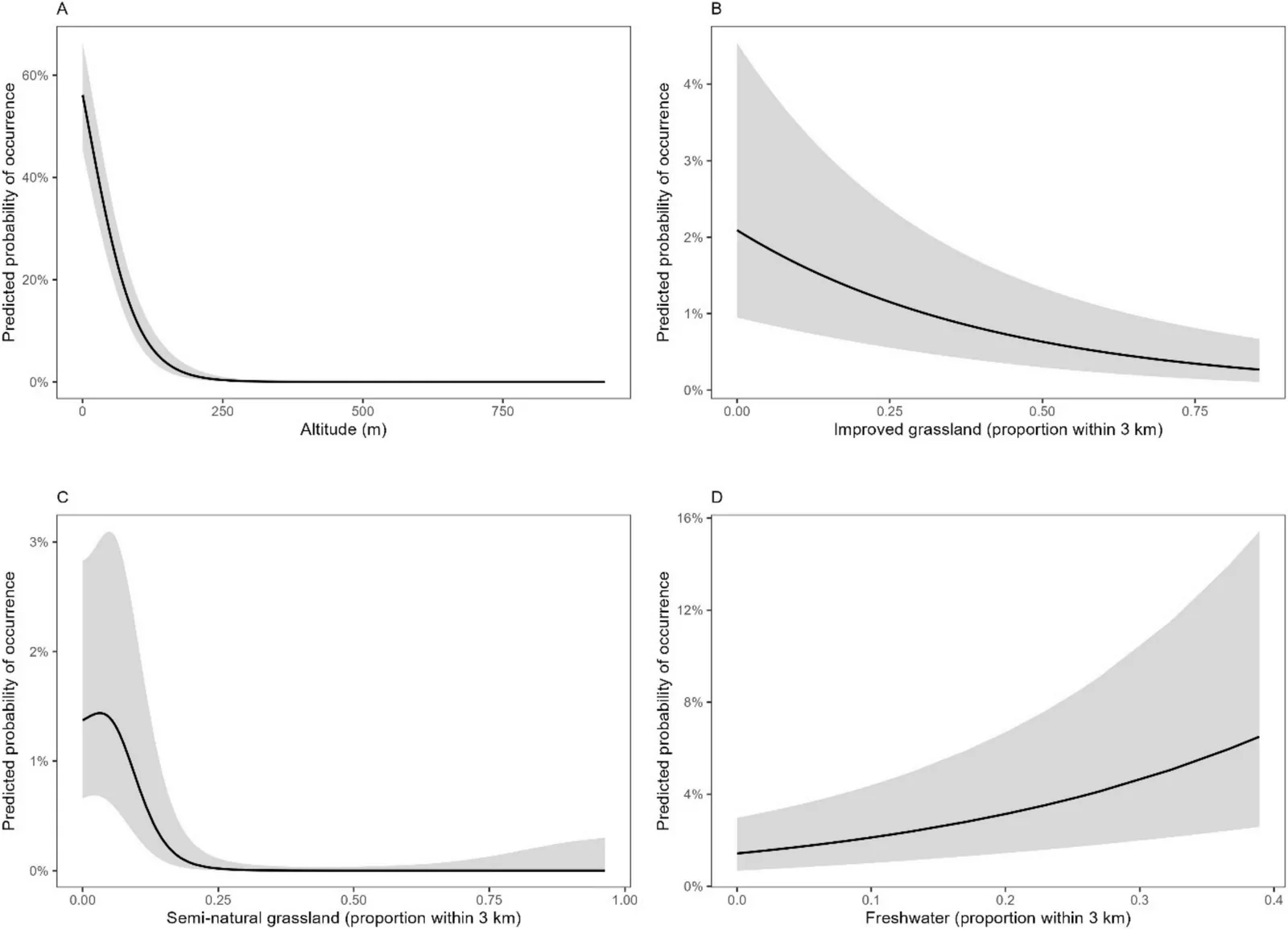
Partial effect plots from the *Anopheles plumbeus* GAM showing the marginal association between predicted response-scale values (interpreted as relative habitat suitability under the presence–pseudoabsence design) and each retained environmental predictor: **A** altitude, **B** improved grassland, **C** semi-natural grassland, **D** freshwater. Solid lines show fitted effects and shaded ribbons indicate 95% confidence intervals

Effect plots (Figs. 2, 3) illustrate how predicted values on the response scale vary along each covariate gradient (interpreted here as relative habitat suitability under the presence–pseudoabsence design). For *An. claviger*, suitability declined steeply with increasing altitude (Fig. 2A), while showing unimodal responses to arable land and improved grassland, with highest suitability at intermediate proportions within the 3 km buffer (Fig. 2B–C). Suitability decreased with increasing saltwater cover (Fig. 2D), while freshwater exhibited a broad unimodal association with highest suitability at intermediate freshwater proportions (Fig. 2E). Climatic effects indicated higher suitability with increased precipitation (Fig. 2F) and increasing suitability at higher temperatures and humidity, with particularly pronounced increases at the upper end of the humidity gradient (Fig. 2G–H). For *An. plumbeus*, suitability likewise declined rapidly with altitude (Fig. 3A). Predicted suitability decreased with increasing improved grassland (Fig. 3B), showed highest values at low levels of semi-natural grassland followed by a rapid decline (Fig. 3C), and increased with increasing freshwater cover (Fig. 3D).

### Predicted habitat suitability across Scotland

**Fig. 4:**
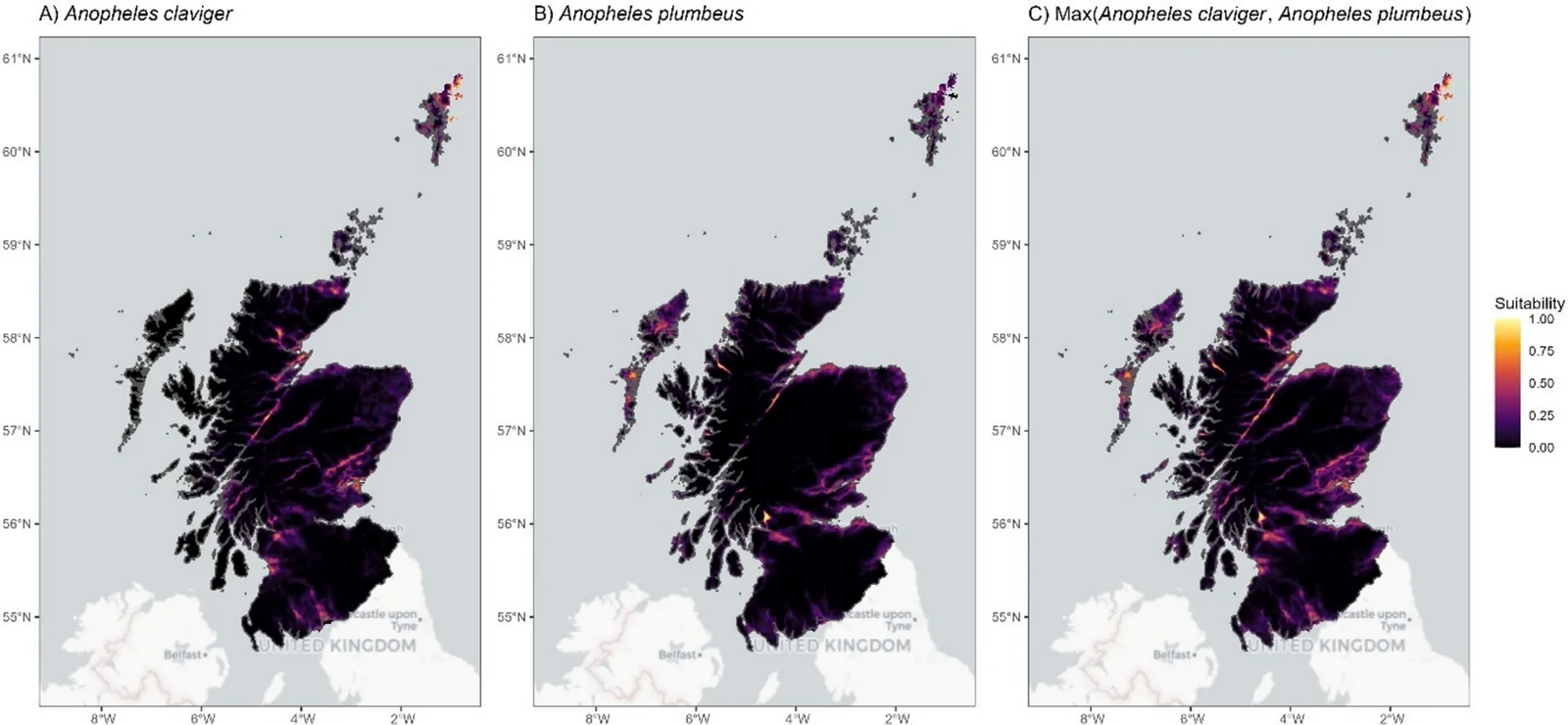
Predicted relative habitat suitability surfaces for **A** *Anopheles claviger*, **B** *Anopheles plumbeus*, and (**C**) the combined surface defined as $\mathrm{m}\mathrm{a}\mathrm{x}({\widehat{p}}_{claviger},{\widehat{p}}_{plumbeus})$, mapped on a common 0–1 response scale

Spatial predictions at 1 km resolution indicated that both focal species had non-uniform predicted suitability across Scotland, with suitability concentrated in discrete clusters rather than being evenly distributed (Fig. 4). *Anopheles claviger* showed comparatively widespread areas of low-to-moderate suitability interspersed with higher-suitability hotspots (Fig. 4A), whereas *Anopheles plumbeus* exhibited a patchier pattern with fewer high-suitability clusters (Fig. 4B). The combined surface (cell-wise maximum of the two species-specific predictions) highlighted areas predicted to be suitable for at least one focal vector; capturing the union of hotspots and expanding the extent of high suitability relative to either single-species map (Fig. 4C).

### Concordance between historical malaria cases and contemporary vector suitability

**Fig. 5:**
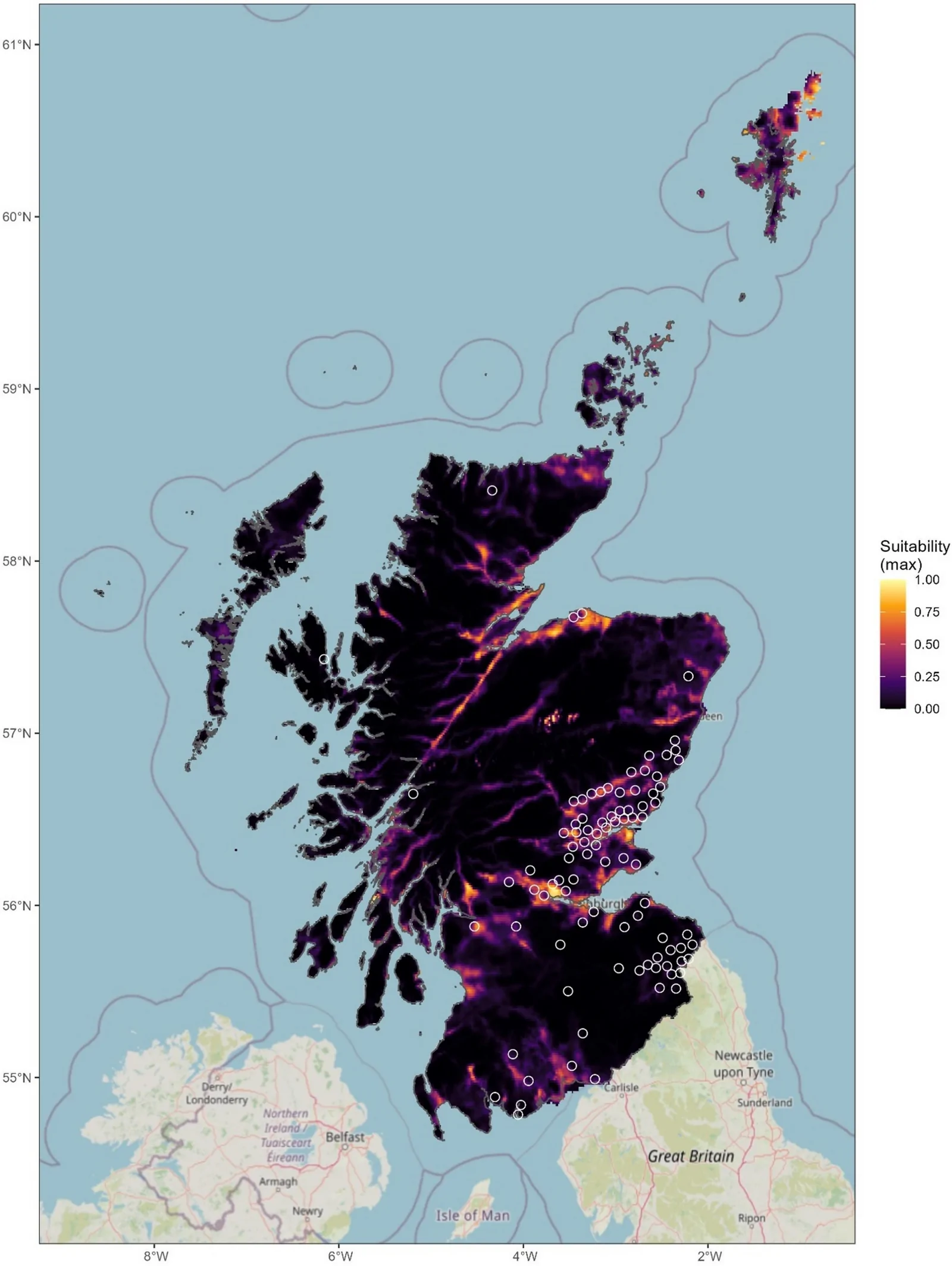
Historical parish-level hotspots of malaria (“ague”) overlaid on the combined contemporary *Anopheles* suitability surface (*An. claviger* and *An. plumbeus*) predicted for Scotland. Points show georeferenced locations of parishes where ague was mentioned as a public health problem in during the 18.^th^ century [36]. The raster surface shows predicted habitat suitability defined as the maximum of the *Anopheles claviger* and *Anopheles plumbeus* suitability predictions (0–1 scale)

Historical parish-level malaria hotspots were consistently associated with higher predicted suitability than background locations, and this spatial pattern is illustrated for the combined maximum suitability surface in Fig. 5.

For *Anopheles claviger*, suitability values extracted at historical hotspots were significantly higher than the background distribution (one-sided Wilcoxon rank-sum: $p=3.65\times {10}^{-8}$), with an AUC-like probability-of-superiority of 0.664. Mean suitability at historical hotspot locations was 0.104, exceeding background expectations under permutation testing (p=0.002). Using the ROC-derived high-suitability threshold for *claviger* (0.180), 18.7% of historical hotspots fell in high-suitability areas compared to 11.0% of background locations (ratio 1.70).

For *Anopheles plumbeus*, the association with historical malaria locations was similarly strong (Wilcoxon $p=3.71\times {10}^{-10}$; AUC-like statistic 0.687). Mean suitability at historical hotspot locations was 0.134, with permutation testing indicating strong support for higher-than-background suitability (p<0.001). Applying the ROC-derived threshold for *An. plumbeus* (0.155), 33.0% of historical cases exceeded the high-suitability cut-off compared to 17.6% of background locations (ratio 1.88).

The combined analysis indicated that historical cases were more likely than expected to occur in areas predicted suitable for at least one focal vector species. Suitability on the combined maximum surface was significantly higher at historical case locations than at background locations (Wilcoxon $p=1.83\times {10}^{-11}$; AUC-like statistic 0.701), with a mean historical suitability of 0.187 (p<0.001 by permutation). When defining “high suitability” using the union of species-specific ROC thresholds $\left( {\hat{p}_{{{\mathrm{claviger}}}} \ge \,0.180{ }\,{\text{or }}\,\hat{p}_{{{\mathrm{plumbeus}}}} \, \ge 0.155} \right)$ 45.1% of historical hotspots fell within high-suitability areas compared to 26.8% of background locations (ratio 1.68).

## Discussion

Here we present contemporary species distribution models and predicted suitability maps of native *Anopheles* species in Scotland, with a focus on two species implicated in historical malaria transmission (*An. claviger, An. plumbeus).* We confirm the broad distribution of these two species across Scotland,, with highest predicted suitability for their occurrence in eastern coastal and lowland regions. We also document the more limited presence of another important historical malaria vector (*An. maculipennis* s.l.). The occurrence of *An. claviger* and *An. plumbeus* was significantly associated with environmental variables including temperature, precipitation, humidity, altitude, freshwater and land-use. Additionally, we show that the contemporary predicted habitat suitability of these vectors aligns closely with qualitatively defined hotpots of malaria spots in Scotland, as derived from eighteenth century historical records.

Our initial goal to develop individual species distribution models for all three historical malaria vector species in Scotland (*An. claviger, An. maculipennis* s.l. and *An. plumbeus*) was not possible due to limited data available for *An. maculipennis* s.l. (n = 5) which were insufficient for spatial prediction. However robust models were obtained for *An. claviger* and *An. plumbeus* and used to identify several physical and environmental covariates predictors of their occurrence. Both species displayed a strong negative correlation with altitude. This is in line with the expectation of high altitudes having cooler, less favourable microclimates for larval development, and similar negative associations with altitude reported for other *Anopheles* species [65, 66]. Hydrology and topography also drive this relationship as runoff water tends to accumulate in large valleys and low lying areas, a phenomenon that concentrates *Anopheles* larval habitats at low altitudes [67]. High altitude areas in Scotland are also largely exposed with little aquatic or mature wooded vegetation, further reducing the quantity and quality of larval habitats. Conversely, lower-altitude areas may also contain more agricultural and settlement-associated features, including livestock-related buildings and empty or abandoned stables, which could provide resting or breeding opportunities for some *Anopheles* species. A recent study in Switzerland reported the presence of *An. plumbeus* in woodlands at elevations well over 1000 m [68], suggesting that the negative association with altitude observed here may be primarily due to differences in vegetation cover.

Land-use variables were also important predictors of presence for both species. For *An. claviger*, unimodal relationships were identified between probability of occurrence and the proportions of arable land and improved grassland, characterised by peak probability at intermediate values (0.3–0.4) and rapid declines toward lower and higher proportions. A possible explanation for these trends lies in the habitat composition within the 3 km buffer from which land-use covariates were extracted. In the UK, natural marshland is frequently bordered by extensive agricultural and grassland ecosystems, potentially placing these land-use types in close proximity to *An. claviger* larval habitats [69, 70]. Intermediate proportions of arable land and improved grassland within the buffer area may therefore be expected as these sites typify marsh habitats embedded within agricultural landscapes. A study conducted in an English fen ecosystem also found that *An. claviger* was most abundant in wetland habitats embedded within a matrix of grass and arable land [43]. In contrast, the negative correlation between *An. plumbeus* probability of occurrence and improved grassland may be explained by both the lack of biodiversity in these habitats (low vertebrate host availability) and their vegetation composition of short ground-ground covering plants. These relatively open habitats may be especially unsuitable to species like *An. plumbeus* that rely on woodland or large trees for larval habitat [71, 72].

Both *Anopheles* species were associated with hydrological variables. The negative association between the probability of *An. claviger* occurrence and the proportion of saltwater is consistent with the larval ecology of this species; characterized by use of inland freshwater larval habitats [21]. The predicted higher *An. claviger* occurrence at intermediate proportions of freshwater can be attributed to their preference for smaller, well vegetated larval habitats which are often absent both at very low and high proportions (e.g. as higher proportions correspond to larger expanses of open water, which are not appropriate larval habitats for this species). The positive association between *An. plumbeus* occurrence and the proportion of freshwater within 3 km likely reflects their known larval ecology and the sampling efforts of the Mosquito Scotland project (through which ~ 70% of *An. plumbeus* observations were derived). *Anopheles plumbeus* is a tree hole breeder and thus strongly related to mature woodland habitat, with trees like oak providing viable larval habitats [28, 72, 73]. A large portion of the *An. plumbeus* observations in this study came from one site, the Scottish Centre for Ecology and Natural Environment, where an ancient oak woodland is situated along the shores of a large lake (Loch Lomond). The concentration of data from this site likely drove the observed relationship with freshwater, with further sampling of oak woodlands near and far to freshwater bodies needed to confirm it.

In contrast to land-use, climatic variables were associated with the probability of *An. claviger* occurrence, but not that of *An. plumbeus*. The presence of *An. claviger* increased with temperature and humidity and peaked at intermediate precipitation levels. Ambient temperature is known to have a strong positive influence on mosquito fitness and abundance up until a thermal maximum, after which there is sharp decline [15, 74, 75]. The positive association detected here indicates that the thermal tolerance limit for this species is unlikely to be exceeded across typical Scottish summer temperatures (6–10 °C in this study). In line with predictions made for other mosquito species in the UK [76], this suggests warming temperatures may increase the abundance of this species. The positive correlation between humidity and *An. claviger* presence is also expected based on previous work showing increased *Anopheles* longevity at higher humidity as a consequence of desiccation risk in arid conditions [77, 78]. The predicted unimodal relationship between *An. claviger* occurrence and precipitation also matches that described for other *Anopheles* species that use similar ground-based larval habitats (e.g. African malaria vectors). In this context, both periods of low or intense rainfall can decrease mosquito populations and larval survival [79–81], due to the lack of larval habitat at one extreme and occurrence of washout at the other. The absence of identified climatic predictors for *An. plumbeus* in Scotland may reflect the broad environmental tolerance of this species. It occurs across a wide range of temperatures in Europe, with capacity to persist through both northern European winters and hot summers in the southern Mediterranean [48, 68]. This species’ use of tree hole larval habitats and capacity to oviposit in artificial containers [73, 82] may also reduce its reliance on precipitation compared to other *Anopheles* species.

Locations of historical malaria hotspots as identified in records from the eighteenth century were predicted to have higher contemporary suitability for *Anopheles* vectors than random locations. A stronger association was observed between historical hotspot locations and the combined suitability surface for both vector species, rather than either vector species on their own. The improved performance of the combined surface is likely because it does not assume a single vector species, instead treating areas as potentially suitable if they support at least one species’ niche. The similar strength of association for each species individually suggests that historical hotspots coincide with conditions suitable for more than one potential vector. This significant overlap between locations of historical malaria and contemporary *Anopheles* distribution indicates that areas that were most suitable for transmission in the nineteenth century may remain so today. Other studies on historical malaria in Northern Europe have found similar patterns, with coastal regions and lowland marshes having the highest rates of historical case incidence [32, 83]. Thus, signatures of historical malaria risk are likely still apparent in contemporary mosquito vector distributions in northern Europe and could be used to identify where the risk of re-establishment is highest. However, the low predicted occurrence of *Anopheles* in some historical hotspots (e.g. south of Scotland) indicate long-term reductions in *Anopheles* habitat suitability has occurred in some regions, likely due to large-scale land-use change including drainage and modification of wetland habitats over the last few centuries.

Historical changes in land drainage are implicated as the primary driver of malaria decline throughout temperate Europe in the nineteenth century [15, 84]. However, these long-term land use changes may be reversed as part of rewilding and restoration programmes. For example, UK wetland restoration and management can create favourable larval habitats for some mosquito species, including potential disease vectors, highlighting the need for site-specific monitoring and integrated management [85]. In Scotland a recent government administration has pledged to restore 400,000 hectares of peatland across the country before 2040 [86]. Peatland restoration occurs by reducing drainage and allowing more standing water to accumulate; a strategy that is very beneficial for increasing carbon capture but that may also increase mosquito larval habitats. Peatland is usually quite acidic [87], so habitat created through these programmes may not be suitable for mosquitoes and thus have no impact on mosquito VBD risk. Additionally, the rich biodiversity and conservation benefits from this restoration may outweigh any smaller and likely localized increased in mosquito nuisance. However further research is needed to understand and mitigate any potential negative consequences of this type of habitat management on mosquitoes.

Several limitations should be considered when interpreting the results of this investigation. Most of the data from the Mosquito Scotland were derived from targeted wetland sampling, thus introducing potential sampling effort biases toward Anophelines with these habitats. Additionally, as records were drawn from different sources and time periods, the data are not fully consistent in how, where, and how intensively mosquitoes were sampled. With a relatively small number of detections, this heterogeneity can influence model fits and means that results should be interpreted most cautiously where model performance was weaker. Finally, the inability to obtain a robust model for *An. maculipennis* s.l. further limited the ability for the study to analyse species specific interactions and gain a full understanding of the distribution of all three malaria vector species present in the region.

This study significantly advances understanding of the ecology of potential *Anopheles* malaria vectors in Scotland through providing an updated prediction of their distribution, and the climatic and physical environmental variables that determine their habitat suitability. We demonstrate that the current distribution of this genus significantly overlaps with regions identified as hotspots of malaria in theeighteenth century, highlighting that these locations may remain a priority for further surveillance of emerging risk in the future. Continued surveillance of these and other potential vector species in Scotland will be crucial to improve the accuracy of vector distribution maps and identify risks of VBD emergence before they become established.

## Data Availability

The dataset used in this study was deposited at VecDyn through the VBD Hub with curation support from Sarah Kelly, and is available for download by request.

## Abbreviations

*An.*: *Anopheles*
UK: United Kingdom
VBD: Vector Borne Disease
